# Collaborative Testing in Medical Education: Insights from a Hybrid Individual–Group Examination Approach

**DOI:** 10.1007/s40670-026-02773-w

**Published:** 2026-06-05

**Authors:** Becca N. Kubick, Andrew R. Thompson, Donald J. Lowrie

**Affiliations:** 1 University of Cincinnati College of Medicine, Cincinnati, OH USA; 2 Department of Medical Education, University of Cincinnati College of Medicine, Cincinnati, OH USA

**Keywords:** Team-based learning, Collaborative testing, Group examination, Retention, Assessment

## Abstract

Team-based learning is a widely accepted instructional approach in undergraduate medical education, but the application of its principles outside of its traditional framework has not been fully explored. Here, we describe the implementation of a hybrid individual-group examination format during a two-week Multisystems course at the University of Cincinnati College of Medicine. Students completed two separate in-house examinations during the course, each with individual and group components, as well as a cumulative NMBE final examination that was taken individually. A portion of the second individual examination retested concepts from the first examination to explore the impact that collaborative test-taking had on student learning. Student perceptions of the experience were evaluated using a survey after the final examination. We found a small but statistically significant decline in average in-house and NBME individual examination scores following the transition from traditional individual testing to the hybrid individual-group format. However, measured improvement in performance on retested concepts suggests that collaborative testing had a positive impact on student learning, especially among lower performing students. This testing model was overwhelmingly supported by students, who reported that group testing enhanced their learning experience and decreased the stress surrounding preparation for the examinations. Opinions on whether this approach to assessment should be utilized other courses were divided and heavily influenced by respondee class rank, highlighting a potential barrier to implementation. These findings shed light on the benefits and drawbacks of collaborative testing in the medical school context.

## Introduction

Scientific inquiry and the practice of medicine are inherently collaborative processes. This understanding has driven numerous initiatives to infuse collaboration into medical school curricula, with the ultimate goal of training physicians who are strong communicators and effective teammates. Team-based learning (TBL), a pedagogy introduced to medical education in the early 2000s, exemplifies this recent shift toward structured, collaborative learning. TBL involves guiding teams of 5–7 students through several phases of learning, including independent study, individual assessment, group re-assessment, and concept application through group learning [1]. Previous studies of TBL suggest that this approach may improve test performance, knowledge retention, and student engagement in the classroom relative to traditional lecture-based instruction [2–4]. Undergraduate medical centers that have incorporated the TBL framework into their curricula have found that student impressions are mixed but skew positive, especially when fidelity to TBL’s core design elements is maintained [5–7]. However, extensive variation in the application of these design elements across institutions has made it difficult to make overarching claims regarding the benefits of this approach over traditional learning models [8–9].

Building on the early successes of TBL, some institutions now seek to incorporate the educational benefits of collaboration into the assessment process itself [10–13]. This is most often sought through implementation of a hybrid testing format that includes a component of student collaboration. For example, students may take examinations individually and then be retested on all or a subset of questions as a group. Collaborative assessment has repeatedly been shown to increase student examination performance across the health professions [10–13]. Evidence of its ability to improve knowledge retention has been less definitive, especially in the specific context of undergraduate medical education. While some studies in the health sciences have demonstrated a significant increase in knowledge retention following collaborative assessment as compared to traditional testing [10, 14–15], others have identified no difference [16–18]. The extent to which collaborative testing affects medical students differently based on their prior academic performance also remains unclear. Past studies have found that group testing typically benefits middle- and lower-achieving learners the most, though all students seem to benefit equally when content is more challenging or questions require higher-order thinking [12, 19–20]. However, studies evaluating the influence of previous academic performance on collaborative testing outcomes are lacking in the medical school context.

The purpose of this study is to explore the effects of a hybrid testing format on medical student performance in a pre-clerkship Multisystems course. By analyzing learning outcomes and student perceptions in the context of class rank, we seek to clarify how collaborative testing affects different learners and explore its practical implications for medical education.

## Materials and Methods

### Study Population

Ethical approval was obtained for this study (University of Cincinnati Internal Review Board, IRB#2025 − 0372). This study involved second-year medical students enrolled in a course titled Multisystems at the University of Cincinnati College of Medicine. This was the last course in the Phase 1 medical school curriculum and included discussion of clinical conditions that affect multiple organ systems, as well as a portion dedicated to review of high-yield topics in microbiology and antibiotic therapy. The two-week course included two separate assessments. The first examination, developed in-house, was administered after one week of lecture content. The second examination, given after the course content was completed, included two separate parts: one part developed in-house, and another assembled and delivered through the National Board of Medical Examiners (NBME) Customized Assessment Service. These questions were intended to serve as a cumulative evaluation of student proficiency in the course. Both examinations consisted exclusively of multiple-choice questions.

**Fig. 1:**
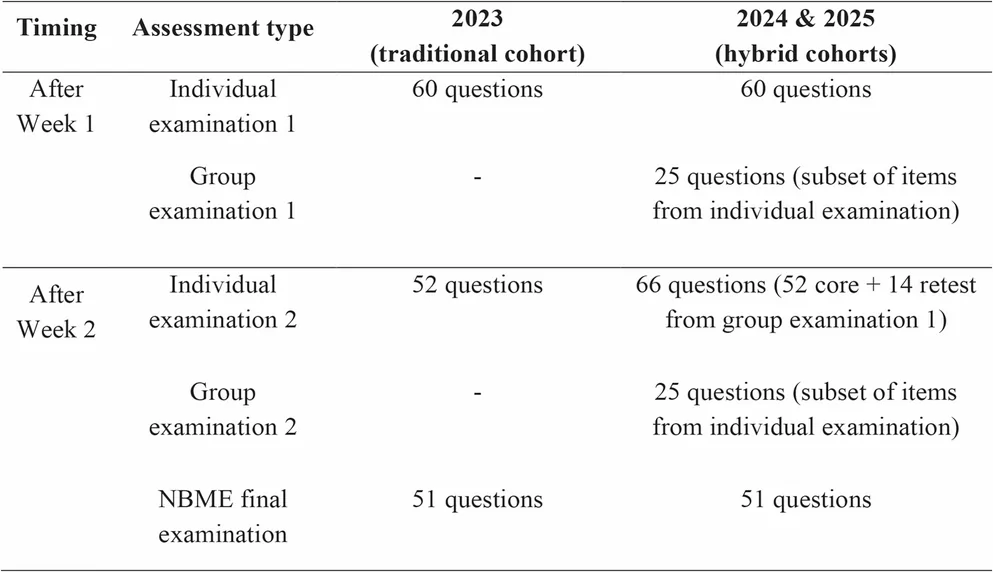
Assessment structure in the Multisystems course. Multisystems is a two-week course. Assessments were administered at the end of each week. In 2023 (traditional testing cohort), the first assessment was a taken individually and consisted of 60 in-house questions. The second assessment had two parts that were both taken individually: the first part consisted of 52 in-house questions, and the second part consisted of 51 questions from the NBME database. For the hybrid testing cohorts in 2024 and 2025, the individual examinations remain consistent, but in-house group assessments were added. These group assessments consisted of the historically most challenging questions asked on the in-house examinations (25 of 60 questions for examination 1; 25 of 52 questions from examination 2). Additionally, the second in-house individual examination for the hybrid testing cohorts included an additional 14 questions that (re)tested concepts from the group examination from week 1. All cohorts took the NBME final examination individually

Three cohorts of medical students were included in this study from 2023 to 2025 (Fig. 1). The first cohort(2023, *N* = 172), referred to as the traditional testing cohort, took the Multisystems course in the traditional format, in which each student completed the assessments individually. Starting with the 2024 cohort (*N* = 170) and continuing in 2025 (*N* = 177), a new assessment strategy was implemented in the Multisystems course. These students, referred to as the hybrid testing cohorts, first took the in-house examinations individually. After a 15-minute break, the students then met with members from their gross anatomy dissection tables (5–6 students in each group) and retook a portion of the same examination as a group. This group examination consisted of a subset of questions from the individual examination that were identified in advance as being “challenging” based on historical data of student performance (< 80% correct in previous years). Students submitted their group examinations separately, providing the opportunity to choose an option that was not the group’s consensus. Students were not permitted to utilize outside resources during any of the examinations. This process of an individual and then group examination was followed for both in-house examinations in the hybrid testing cohorts. Lastly, students individually completed the NBME final examination.

## Study Design

The goal of the present study was to evaluate the impact this hybrid individual/group testing format had on student learning and experience, as indicated by assessment performance and reported student satisfaction. To assess differences in performance between cohorts, we utilized individual scores on both in-house and NBME questions that were asked identically in each of the three years. There were a total of 60 questions on the first in-house examination, 52 questions on the second, and 51 questions on the NBME summative assessment that met this criterion (Fig. 1). The group examinations administered in 2024 and 2025 were constructed using the same subset of 25 questions from that week’s individual assessment using the criteria described above. For the traditional testing group, the first examination was weighted as 40% of their grade, the second examination was weighted at 30%, and the NBME final was 30%. For the hybrid testing groups, all five examinations (two individual, two group, and one NBME) were equally weighted at 20%.

To gauge how the group testing experience impacted student learning, 14 new questions covering content from the first examination were included on the individual portion of the second examination for the hybrid testing cohorts. The questions tested concepts on which students performed poorly individually but discussed with their team during the subsequent group examination. While they tested the same concepts, these were entirely new questions. They were written at a similar difficulty level, but had a completely different question stem with a correct answer that was different from that of the original question. These new questions were then included in the second individual examination without advance notice given to students. Individual performance on these questions allowed us to approximate the effect the hybrid testing structure had on student learning and short-term retention.

In addition to general, cohort-wide comparisons, we were also interested to see if the hybrid testing model differentially impacted students based on their previous history of academic performance. At the time of the study, the University of Cincinnati College of Medicine utilized a quartile ranking system during Phase 1 of the curriculum. Class rank was determined based on course grade averages, which were weighted by the number of credit hours assigned to each course. The class rank used in this study was based on the quartile rank recorded for each student at the completion of Phase 1. It is important to note that approval of the hybrid examination model was contingent on the Multisystems course being removed from the calculation of class rank. Thus, for students in the traditional examination cohort, Multisystems did contribute to class rank (albeit a minor contribution, as it was a short, three credit hour course), whereas Multisystems was not included in the class rank calculation for the hybrid examination cohorts.

To assess student satisfaction, feedback on the hybrid testing model was obtained via survey at the completion of the second group examination in 2024 and 2025. The survey questions were designed to gather general impressions on the hybrid testing model and evaluate whether it could be utilized in other courses within the Phase 1 curriculum. Likert scale results of the survey were dichotomized into “agree” and “disagree” categories to ease interpretation.

## Statistical Methods

Comparisons of student performance between cohorts were made using Analysis of Covariance (ANCOVA) with Medical College Admissions Test (MCAT) scores as a covariate. This was done to account for potential baseline differences in student aptitude between cohorts. We chose to use this as a covariate since it is a standardized test with scores that could be applied consistently across all cohorts in this study. Effect size is reported as eta-squared (η^2^), which is calculated by dividing the sum of squares between groups by the total sum of squares (Levine and Hullett, 2002). Interpretation of these values follows Cohen’s 1988 guidelines for small (η^2^ = 0.01–0.06), medium (η^2^ = 0.06–0.14), and large (η^2^ ≥ 0.14) effect sizes. Tukey’s HSD post-hoc test was used to evaluate pairwise differences between cohorts. Although there were two sets of in-house individual examinations for all cohorts, the results for each cohort were combined into a single average in-house examination score for all comparisons. All analyses were conducted using SPSS version 27 (IBM Cort., Armonk, NY).

## Results

**Fig. 2:**
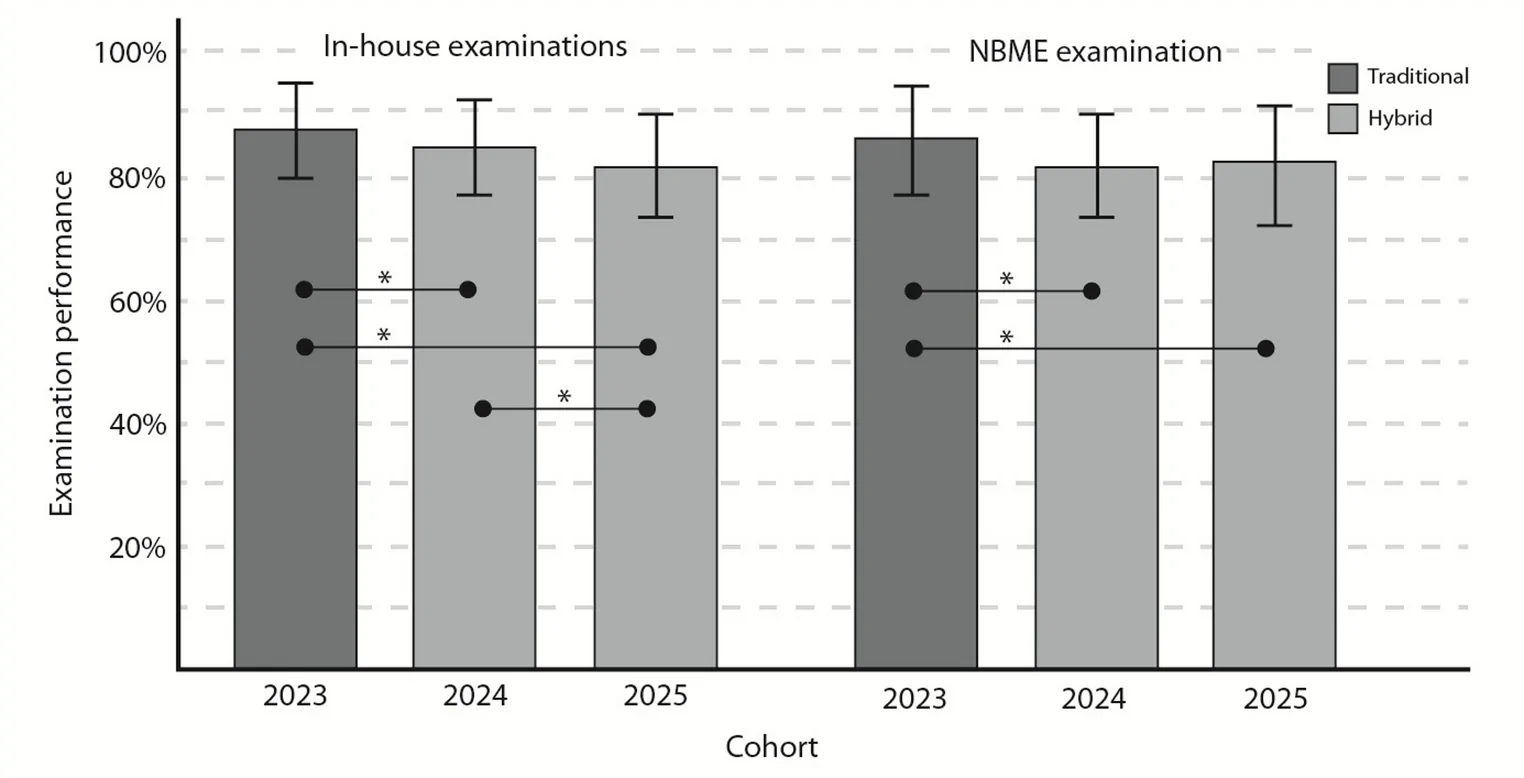
Student performance on in-house and NBME examinations. Students in both the traditional (2023) and hybrid testing (2024 and 2025) cohorts completed two individual in-house examinations consisting collectively of 112 questions. The average percent correct across both assessments was recorded for each cohort. Students also completed a 51-question NBME examination at the end of the Multisystems course; the average score for each cohort is recorded. Error bars represent +/- 1 standard deviation. Statistically significant (*p* ≤ 0.05) pairwise comparisons are noted by an asterisk (*)

Comparing scores on in-house and NBME examinations revealed a decrease in individual student performance following implementation of the hybrid testing format (Fig. 2). The overall average on in-house examinations in 2023 was 86.9%, compared to 83.7% in 2024 and 81% in 2025. The difference was statistically significant (*p* < 0.001) with a medium effect size (η^2^ = 0.081). This pattern carried through to the NBME final examination, where students in the traditional examination cohort averaged 85% compared to 81.2% and 81.5% for years 2024 and 2025, respectively. The difference was statistically significant (*p* < 0.001) with a small effect size (η^2^ = 0.036). Post-hoc tests revealed significant differences between all three cohorts on the in-house examinations. NBME examination scores differed significantly between students in the traditional examinations cohort and both hybrid examination cohorts, but there was no significant difference in scores between the two hybrid cohorts.

To determine if the hybrid testing format differentially impacted students based on their past performance, examination results were reassessed in the context of class rank (Table 1). We found that hybrid testing students in all four quartiles scored significantly lower on in-house examinations than rank-matched traditional testing students. The greatest difference in performance was observed among students in the lower three quartiles, where the effect sizes were large (η^2^ = 0.175–0.271). Summative NBME scores also declined across all quartiles under the hybrid testing model. The difference was again more notable among students in the second, third, and fourth quartiles; the effect sizes were medium (η^2^ = 0.098–0.114).

**Table 1:** In-house and NBME examination performance stratified by class rank. Each cohort was divided into four groups based on their class quartile ranking at the beginning of the Multisystems course. Average examination performance for each quartile on both in-house and NBME examinations is recorded

	In-house examinations				
	2023	2024	2025	Sig	η^2^
Q1	93.4	90.1	90	0.003	0.095
Q2	90.5	86.1	83.7	< 0.001	0.231
Q3	86.8	82.4	78.3	< 0.001	0.271
Q4	79.2	74.1	71.4	< 0.001	0.175
	NBME final examination				
	2023	2024	2025	Sig	η^2^
Q1	92.1	89.9	90.1	0.046	0.05
Q2	87.3	82.3	84.7	< 0.001	0.114
Q3	84.5	79.1	78.9	0.001	0.098
Q4	78.7	72.7	71.7	0.001	0.114

To further elucidate the impact of group testing, we next compared student performance on questions from the individual examinations that were reassessed during the group portions. Unsurprisingly, average scores were significantly higher in the group format. On average, students answered 70% of the questions correct individually, compared to 96% as a group; this improvement was statistically significant (*p* < 0.001).

**Fig. 3:**
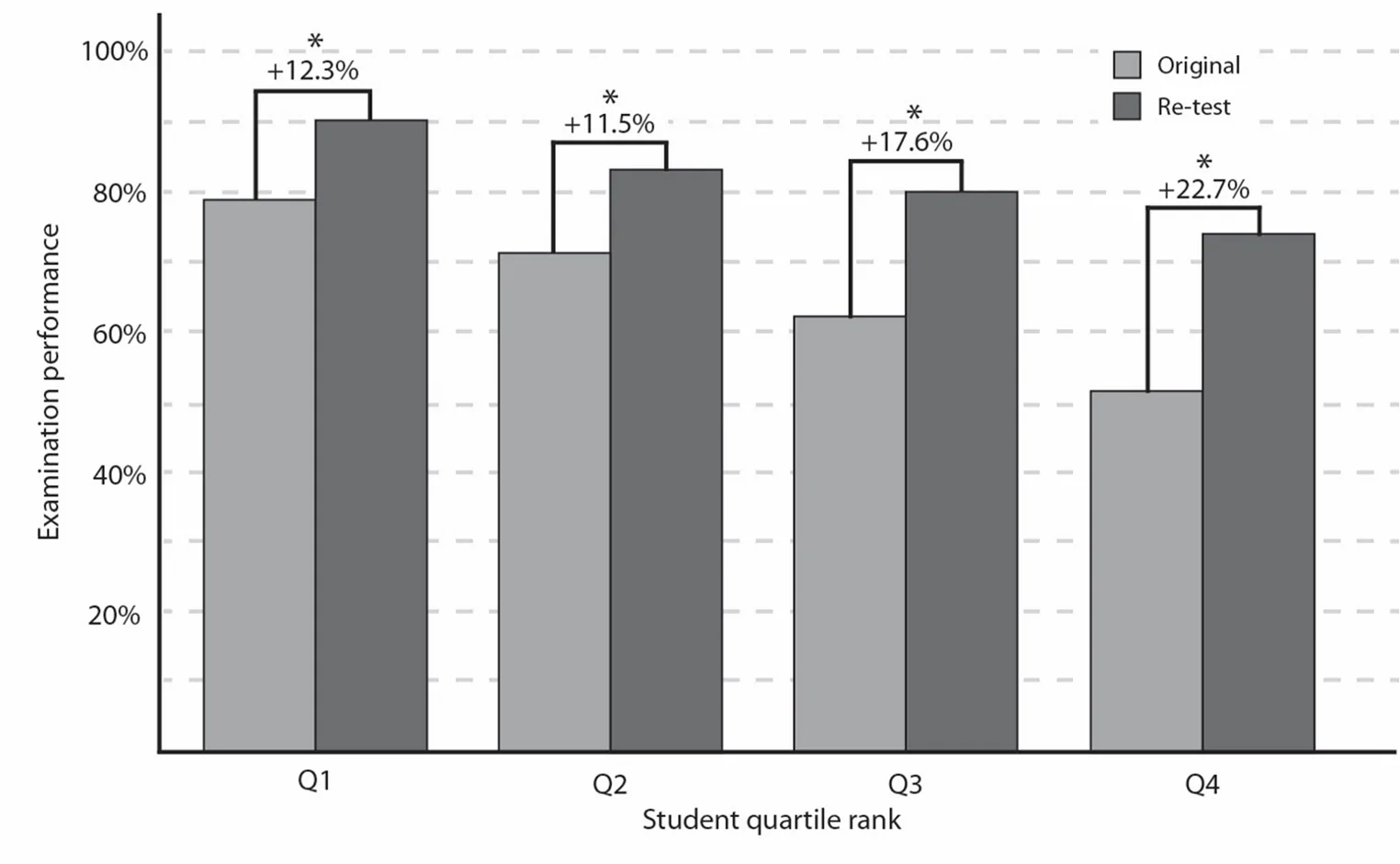
Percent improvement on retested concepts by class rank. Both hybrid testing cohorts were administered 14 questions on their second individual examination that retested concepts from the first examination. For students in each of the class rank quartiles, average scores on these questions for the first examination and the retesting are compared, with their percentage improvement indicated above. The two hybrid testing cohorts were combined for this analysis. Statistically significant (*p* ≤ 0.05) differences are noted by an asterisk (*)

To gauge the degree to which this hybrid testing structure facilitated learning, challenging concepts that were included as part of the first group examination were retested using new questions on the second individual exam. Overall, students performed significantly better (*p* < 0.001) on the retesting (82%) compared to the original testing of the same concepts (66%). Stratifying these results by class rank revealed significant improvement across all quartiles (*p* < 0.001), though to varying degrees (Fig. 3). Post-hoc comparisons demonstrated that the degree of improvement was greater among students in the third and fourth quartiles compared to those in the first and second quartiles (*p* < 0.001–0.019).

**Fig. 4:**
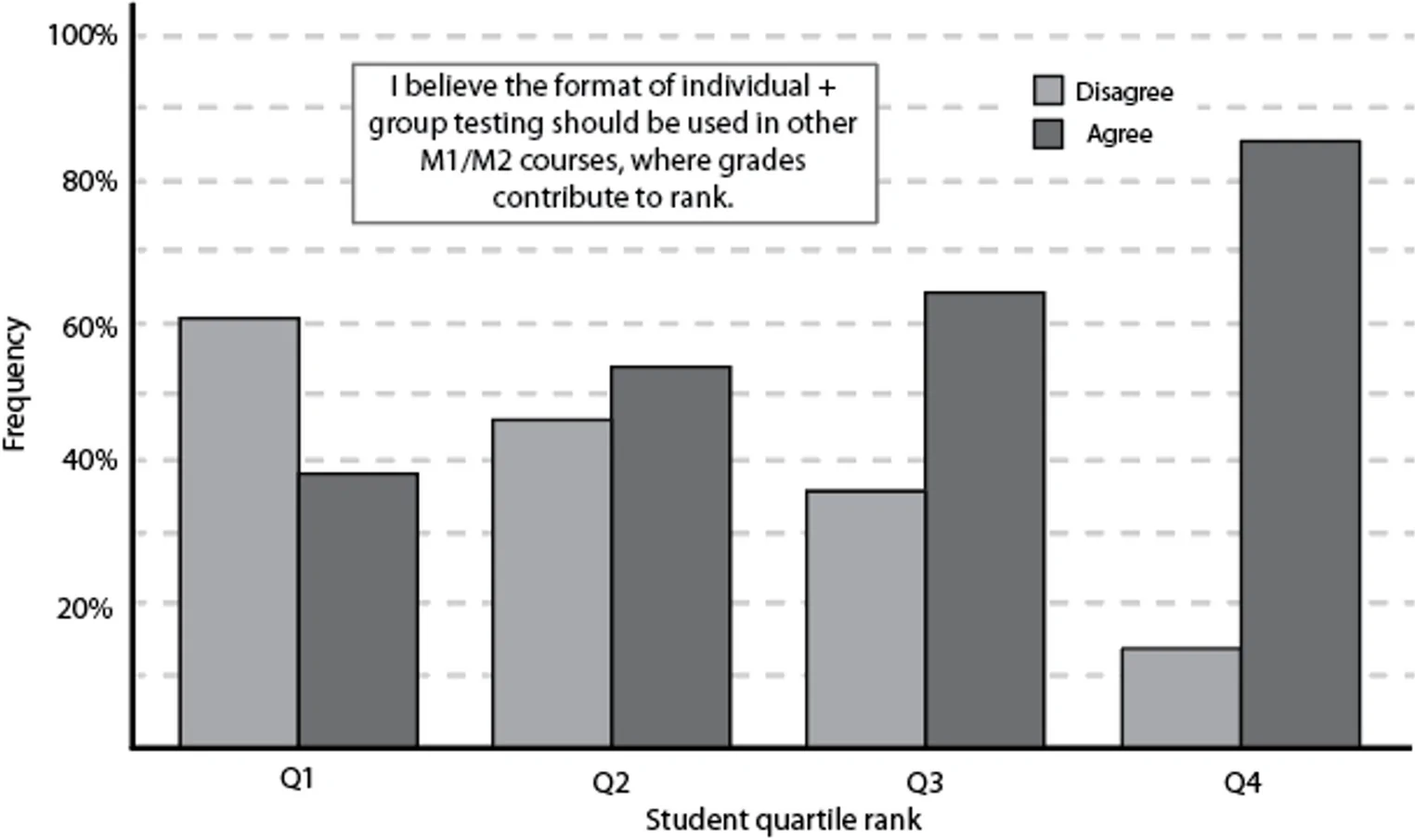
Survey Question 3 responses stratified by class rank

Student feedback on the hybrid testing format was collected following the final group examination of the Multisystems course. Because participation was required, 100% of students responded (*N* = 347). Overall, student satisfaction with the hybrid testing format was overwhelmingly positive (Table 2). Only Question 3, which asked whether this testing format should be used in other courses where grades contribute to class rank, had mixed feedback, with 40.3% of students disagreeing with this statement. To further explore the feedback from this question, responses were grouped according to class rank (Fig. 4). Stratification revealed that students in the higher-performing quartiles, particularly the first quartile, were most likely to disagree with the statement. In contrast, students in quartiles three and four were more likely to agree that the hybrid testing format should be used in other courses.

**Table 2:** Student survey feedback on collaborative testing

	Survey question	Disagree	Agree
1	I like the group exams.	2.4%	97.6%
2	My learning was enhanced by the group exams and discussions.	3.6%	96.4%
3	I believe the format of individual + group testing should be used in other M1/M2 courses, where grades contribute to rank.	40.3%	59.7%
4	If I was unsure about a question, I felt comfortable asking my group to explain their rationale for a particular answer.	0.3%	99.7%
5	I felt comfortable sharing my own rationale for answers to questions with my group.	1.2%	98.8%
6	My group’s dynamic allowed opportunity for all group members to contribute to discussions.	0.6%	99.4%
7	I felt less stressed preparing for these exams because of the group format.	9.9%	90.1%

## Discussion

This study sought to explore the effect a hybrid individual/group testing format had on student examination performance and learning in the medical school setting. Unsurprisingly, students in the hybrid examination cohorts scored significantly higher on the collaborative portions of their examinations than on the individual portions, which is consistent with previous findings [11–12, 19–20]. However, we found that students in the traditional examination cohort performed significantly better than the hybrid cohort students on individual portions of both weekly in-house examinations and on the NMBE final. It is important to note that the effect size of this difference in performance was larger on biweekly examinations (η^2^ = 0.08, 0.064) than on the cumulative final (η^2^ = 0.036). The summative final is where we hoped the benefits of group testing would be most evident since this is where the learning that occurred during group examinations could be applied. While the observed decline in performance on the NMBE final was statistically significant, the small effect size may suggest limited practical implications of the difference. In fact, when the performance difference on the summative final is converted to actual questions, the hybrid cohort students missed an average of < 2 questions more compared to students in the traditional examination cohort.

Considering the changes made to the grading criteria in the Multisystems course between the traditional and hybrid testing groups, the decrease in the hybrid testing cohorts’ examination averages was not entirely surprising. For students in the traditional examination cohort, the Multisystems course grade contributed to class rank, whereas Multisystems was removed from the class rank calculation for the hybrid testing cohorts. In addition, the group examinations used in the hybrid testing cohorts were weighted rather heavily (overall, 40% of their final grade), which provided a considerable grade cushion. Both of these factors may have influenced how much some students prioritized studying for the course, especially since many were beginning to shift focus to the USMLE Step 1 examination.

Repeating the analyses of performance on individually answered questions with students quartered by class rank allowed us to determine whether a specific subset of the cohort was disproportionately affected by the shift in testing strategy. Interestingly, though rank-matched performance was lower in the hybrid examination cohorts across all four quartiles, the difference among students in the top quartile were relatively modest. Top-quartile hybrid cohort students scored roughly 3% lower on weekly examinations and 2% lower on the cumulative final compared to top-quartile students from the traditional cohort. Lower-quartile hybrid cohort students, in contrast, scored 5–7% lower on weekly examinations and 4–7% lower on the cumulative final than lower-quartile students in the traditional cohort. One possible explanation for this disparity relates to preparation for the USMLE Step 1 board examinations. The Multisystems course, which included several high-yield board examination topics, immediately preceded the Step 1 dedicated study period. While there is no specific data to support this idea, it is possible that higher-performing students were further along in their preparation for Step 1 and were thus more likely to be familiar with the Multisystems course content.

While many endorse the educational benefits of group testing and enjoy the experience, some students in this study expressed concern about using this testing format in courses that contribute to class rank. Group testing in any format is often seen as disproportionately improving the grades of low-performing students, based on the assumption that their scores would be bolstered by their high-performing peers without reciprocal benefits [21–23]. Post-examination surveys administered to the students in the hybrid cohorts suggest that these beliefs were pervasive. Students overwhelmingly felt that collaborative testing enhanced their learning, promoted valuable discussions about answer rationale, and decreased stress surrounding examination preparation. Survey responses were largely supportive of the hybrid testing model, until students were asked whether it should be implemented in courses where examination scores contribute to rank. Only 60% of students agreed with this claim, while all other questions boasted more than 90% agreement. Stratification of survey responses by class rank revealed the strong influence of respondee rank on their feelings toward collaborative testing in ranked courses. Most first-quartile students disagreed that collaborative testing should be extended to courses influencing rank, while 85% of fourth-quartile students believed it should. Other studies that found students in higher education to be supportive of broader implementation of collaborative testing in their curriculum [10, 24] were not conducted on cohorts subject to a rank system. Pitting students against each other through rank may beget certain perspectives on the drawbacks of cooperation that are not conducive to utilization of collaborative testing. However, the hybrid individual/group model may circumvent this concern to an extent if individual examination performance is weighted heavily.

While it is important to consider how collaborative testing affects student examination scores, especially in the context of a rank system, its effect on student learning warrants equal attention. We assessed this parameter by extracting challenging concepts from the first group examination and retesting them using new questions on the second individual examination, without alerting the students ahead of time. While students from all four quartiles demonstrated a significant improvement in performance on these questions, the largest margin of improvement was observed in the third and fourth quartiles. Individuals in these quartiles improved significantly more compared to students in the first and second quartiles. This outcome aligns with Lindsley et al. [25], who reported that, although overall retention between traditional and hybrid examination models did not differ, the hybrid model did significantly improve retention of items which students initially missed. Our study supports this conclusion, suggesting that collaborative testing may boast specific utility in reinforcing concepts that were not mastered the first time. While we presume that group testing played an important role in the observed increase in retest performance, it is important to note that we could not fully explore this since the retest approach was not used in the traditional testing group.

It is interesting that, while students in lower quartiles had more room for individual improvement, students in the top quartile did not reach the ceiling (i.e., 100% correct). This suggests that group testing may have utility in decreasing the knowledge disparity between higher and lower-performing students, which ultimately aligns with a fundamental goal of medical education: not to stratify learners, but to cultivate competent, confident, and well-prepared physicians. However, it is simultaneously important to recognize that medical schools must be able to confidently affirm the competency of each individual student, a task which canonically has been primarily accomplished through analysis of assessment individual performance. There are certainly threats to patient safety if a student’s competence is misjudged. Any curriculum seeking to incorporate collaborative assessment should maintain avenues for analysis of individual student competence.

To contextualize the advantages and disadvantages of collaborative testing presented here, it is important to consider the potential logistical barriers associated with the implementation of a hybrid group/individual testing model in medical education. Determination of group size, for example, can have a significant impact on interaction characteristics and therefore the degree of learning that takes place during a group examination. Previous studies have shown that increasing group size can reduce the amount of communication between members and increase the degree of social loafing, lessening individual accountability and potentially impeding discussion [21, 26–28]. At the same time, groups that are too small may lack the diversity of perspectives necessary to stimulate meaningful discussions that promote learning. Complications related to small group activities such as group testing come to a head when considering classes with large (150+) enrollment where orchestrating collaborative tests involves finding private physical space for 20–30 groups, creating time in the curriculum for these lengthier examinations to occur, and determining what criteria to use in assembling the groups to best facilitate the learning experience. Given the student survey results presented here, medical schools utilizing a ranked grading system in the pre-clerkship phase may find more hesitation among students to freely collaborate in a group testing environment.

In an ideal setting, this study would have utilized control groups within the hybrid testing cohorts that were assessed by traditional testing to more directly evaluate whether collaborative testing has a comparatively stronger effect on student learning and retention. Cortright et al. [10] utilized this format in the context of an undergraduate exercise physiology course and found that students demonstrated significantly higher retention on individual repeat examinations when they completed the original examinations in groups as opposed to individually. However, similar studies have found no difference in retention between the two testing formats [16–17]. Lindsley et al. [25] observed improved retention with the hybrid model specifically for items that students initially answered incorrectly, suggesting that collaborative testing may have particular utility in correcting misunderstandings as opposed to broadly solidifying the student knowledge base. Since our study was somewhat opportunistic in nature, there was no possibility of creating a control group. Future work utilizing a more controlled setting could help evaluate the comparative effect of hybrid testing on student learning and retention, specifically in the medical school context.

### Limitations

The present study was performed at a single academic institution and was limited to three cohorts of students, which inherently restricts the generalizability of its findings. Differences in institutional culture, curricular design, examination structure, and approach to student stratification/grading may influence how hybrid testing models are perceived or impact performance at other medical schools. Additionally, with only three cohorts examined, the ability to detect long-term trends is constrained. Unfortunately, changes in the curricular structure preclude the ability to collect additional years of data.

As previously stated, approval of the hybrid examination model was contingent on the Multisystems course being removed from the calculation of class rank. This creates a confounding factor in our study, as the Multisystems course was included in the rank calculation for the 2023 cohort but had no effect in 2024 or 2025. The incentive to maintain or improve rank may have motivated students in the 2023 cohort to approach assessments more earnestly relative to students in later cohorts, who may have perceived less at stake. That said, the impact of this difference was likely minimal, as Multisystems was a short course and thus made a relatively minor contribution to overall class rank.

## Conclusion

Collaboration and a strong foundation of medical knowledge are essential competencies in medical practice and therefore must be valued when constructing medical school curricula. Team-based learning has been widely studied and is common in medical education, whereas the incorporation of a group assessment model is less frequent. Given previous findings that collaborative testing may enhance student learning and engagement, we sought to evaluate its impact in the context of a pre-clerkship medical school course. Students in the lower rank quartiles seem to benefit the most from this assessment model, demonstrating the greatest degree of improvement on retested concepts following their participation in group testing. While a reduction in individual examination scores following the transition from traditional (individual) testing to a hybrid (individual-group) testing format was found across all examinations, it was most pronounced on in-house examinations and less so for the NMBE final examination. Despite this, students clearly perceived the group examination format as beneficial. This feedback, in conjunction with the negligible impact on cumulative final examination results and measured improvement on retested concepts, makes a case that collaborative testing should be considered when designing assessment strategies in the context of medical education.
